# *Trypanosoma cruzi*: Entry into Mammalian Host Cells and Parasitophorous Vacuole Formation

**DOI:** 10.3389/fimmu.2013.00186

**Published:** 2013-08-01

**Authors:** Emile Santos Barrias, Tecia Maria Ulisses de Carvalho, Wanderley De Souza

**Affiliations:** ^1^Laboratório de Ultraestrutura Celular Hertha Meyer, Instituto de Biofísica Carlos Chagas Filho, Universidade Federal do Rio de Janeiro, Rio de Janeiro, Brazil; ^2^Laboratório de Biologia, Instituto Nacional de Metrologia, Qualidade e Tecnologia – Inmetro Duque de Caxias, Rio de Janeiro, Brazil

**Keywords:** *Trypanosoma cruzi*, mammalian cell, endocytosis, phagocytosis, active penetration, host cell, interaction

## Abstract

*Trypanosoma cruzi*, the causative agent of Chagas disease, is transmitted to vertebrate hosts by blood-sucking insects. This protozoan is an obligate intracellular parasite. The infective forms of the parasite are the metacyclic trypomastigotes, amastigotes, and bloodstream trypomastigotes. The recognition between the parasite and mammalian host cell, involves numerous molecules present in both cell types, and similar to several intracellular pathogens, *T. cruzi* is internalized by host cells via multiple endocytic pathways. Morphological studies demonstrated that after the interaction of the infective forms of *T*. *cruzi* with phagocytic or non-phagocytic cell types, plasma membrane (PM) protrusions can form, showing similarity with those observed during canonical phagocytosis or macropinocytic events. Additionally, several molecules known to be molecular markers of membrane rafts, macropinocytosis, and phagocytosis have been demonstrated to be present at the invasion site. These events may or may not depend on the host cell lysosomes and cytoskeleton. In addition, after penetration, components of the host endosomal-lysosomal system, such as early endosomes, late endosomes, and lysosomes, participate in the formation of the nascent parasitophorous vacuole (PV). Dynamin, a molecule involved in vesicle formation, has been shown to be involved in the PV release from the host cell PM. This review focuses on the multiple pathways that *T. cruzi* can use to enter the host cells until complete PV formation. We will describe different endocytic processes, such as phagocytosis, macropinocytosis, and endocytosis using membrane microdomains and clathrin-dependent endocytosis and show results that are consistent with their use by this smart parasite. We will also discuss others mechanisms that have been described, such as active penetration and the process that takes advantage of cell membrane wound repair.

## Introduction

**Figure 1:**
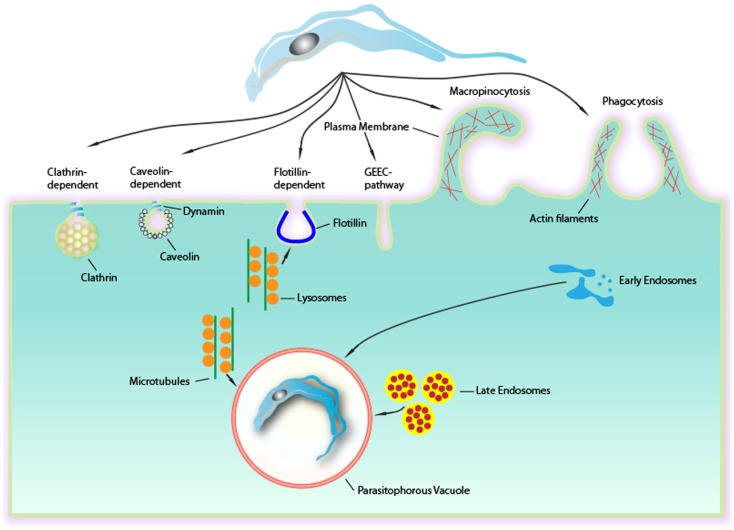
**Endocytic mechanisms involved in *Trypanosoma cruzi* entry into mammalian cells can occur via several different mechanisms culminating in a formation of a PV**. Although phagocytosis was the first endocytic mechanism described to be used by *T*. *cruzi*, others mechanisms as clathrin-mediated endocytosis, caveolar-dependent, and lipid raft-dependent endocytosis macropinocytosis seems to be involved. Formation of the PV is always depends on lysosomes. This fusion can occur at the site of entry of the parasite or after entry, with the PV preformed. The fusion of lysosomes in areas of entry-dependent flotillin was recently demonstrated, but it is believed that this can occur in other ways. The targeting of lysosomes to entrance region or the PV occurs via microtubules. Upon entry there is also the fusion of endocytic vesicles (endosomes and late initials) that together with the fusion of lysosomes leads to the maturation of the PV through their acidification. This allowed the destruction of this maturing vacuole of the parasite to escape later.

*Trypanosoma cruzi*, the causative agent of Chagas disease, is an obligatory intracellular parasite that belongs to the Kinetoplastida order, and it is recognized by the WHO as one of the world’s 13 neglected tropical diseases, affecting 16 million people in Latin America. After the initial infection by the parasite, some patients can develop acute signs and symptoms, including fever, hepatosplenomegaly, and inflammatory reactions. These acute symptoms can be spontaneously resolved. However, the majority of patients are asymptomatic. After the acute phase, a symptomatic chronic form can develop 10–20 years after the initial infection, causing irreversible damage to the heart, esophagus, and colon, with severe disorders of nerve conduction in these organs. Therefore, Chagas disease is characterized as a chronic, systemic, and endemic disease affecting approximately 16 million in Latin America (1) and is considered the major parasitic disease burden of the American continent (2). This parasite presents a complex life cycle that occurs in both vertebrate and invertebrate hosts, where three major developmental stages are observed: epimastigotes, trypomastigotes, and amastigotes. The infective forms of *T. cruzi* (amastigotes and trypomastigotes) are able to infect a wide range of nucleated mammalian cells. The intracellular cycle can be divided into several steps and begins when the infective forms attach and are recognized by the host’s cell surface (3). Then, cell signaling processes lead to the internalization of the parasite in a process that involves the formation of an endocytic vacuole known as the PV. This review will focus on several processes that have been shown to be involved in the internalization of *T. cruzi*, such as phagocytosis, active entry, endocytosis dependent on membrane microdomains (flotillin- and caveolin-dependent), endocytosis mediated by clathrin and macropinocytosis (Figure 1).

## Recognition between *Trypanosoma cruzi* and the Mammalian Host Cell: A Mechanism Dependent on Receptors and Ligands

**Figure 2:**
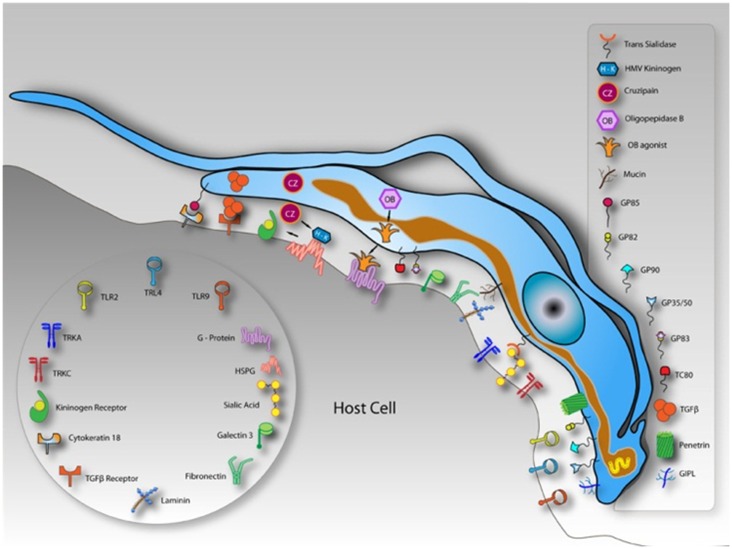
**Schematic model demonstrating molecules involved on parasite-host cell interaction process and exposed on the surface of a hypothetical host cell and in trypomastigotes of *Trypanosoma cruzi***. After Ref. (3).

Classically, the interaction between host cells and *T. cruzi* has been divided into two different steps: adhesion (which includes recognition and signaling) and internalization (3). The internalization process is described as occurring through several pathways that resemble endocytic mechanisms. These two steps are easily distinguished because interactions performed at 4°C do not allow parasite internalization and the parasites remain attached to the host cell plasma membrane (PM), suggesting that the internalization process only occurs at higher temperatures (higher than 18°C) (4). Endocytic mechanisms control the lipid and protein composition of the PM, thereby regulating how cells interact with their environments (5). Endocytosis creates an essential interface between eukaryotic cells and their surroundings through the formation, budding, and maturation of PM-derived intermediates. That endocytosis comprises a sophisticated array of different pathways is now widely accepted (6). Mechanisms involved in cellular uptake are important for different processes in a wide variety of cell types. Classically, these mechanisms can be classified into a number of clathrin-independent pathways as well as clathrin-mediated endocytosis (CME), caveolae, phagocytosis, macropinocytosis, and circular dorsal ruffles (5). Additionally, pathogens often exploit endocytic routes to mediate their internalization into cells (7, 8). Although several studies have been conducted in the field of pathogen and host cell interactions, the molecular mechanisms, including the types of endocytic pathways and the proteins involved in cargo recruitment and internalization, are not completely clear (7). Actually, endocytic pathways start with the recognition between the molecules present and exposed on the cell surface and the product that will be internalized (7). Several *T. cruzi* molecules have been described as being involved in the process of invasion. One class of these molecules is the mucins, which are major *T. cruzi* surface glycoproteins (7). Many mucins have been reported as *T. cruzi* ligands because their sugar residues interact with mammalian host cells (9–12). Other *T. cruzi* molecules involved in adhesion are trans-sialidases (active and inactive) and glycoproteins (gp82, gp80, gp35/50, and gp85) (13). With respect to the mammalian host cell, any class of molecules exposed on the host cell surface is believed to have the potential to be a *T. cruzi* receptor ligand. Most of the characterized receptor classes are carbohydrates that contain galactosyl, mannosyl, and sialyl residues (3, 14–19) and lectin-like proteins, such as galectin 3 that bind to carbohydrate residues present on the parasite surface (20–22). Some lectins, as mannose binding lectin, are involved in a humoral pattern-recognition molecule important for host defense. In the case of Chagas’ disease this lectin is involved in regulating host resistance and cardiac inflammation during infection (23). Other molecules that function as receptors is possibly involved in the pathogenesis of Chagas’ disease are endothelin 1 and bradikinin receptors. They are used by tripomastigotes to invade cardiovascular cells leading to a chagasic vasculopathy (24). Cytokeratin 18, fibronectin, laminin, and integrins are also receptor molecules because the Tc85 present on the trypomastigote surface has motifs that bind to these molecules, making a bridge between the parasite and the host cell (Figure 2) (25–27). We will not describe all the putative molecules involved in *T. cruzi*-host cell interactions because this topic has been discussed in recent reviews (3, 28) and will be covered by other authors in this issue.

## Phagocytosis

**Figure 3:**
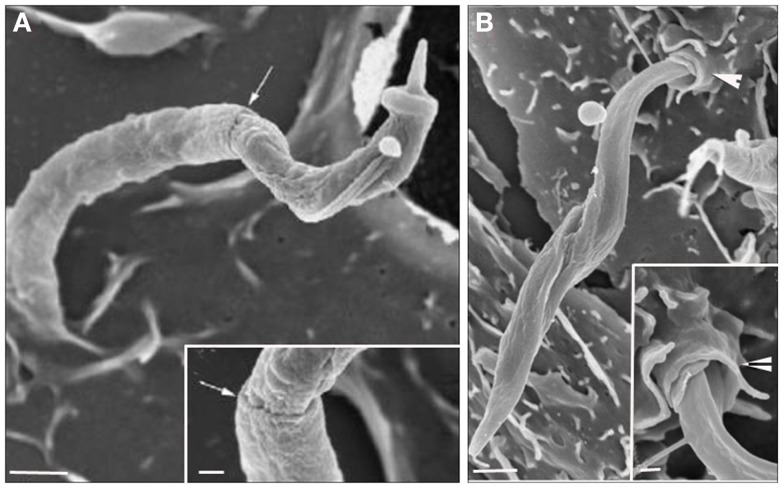
**Field emission scanning electron microscopy of the interaction between peritoneal macrophages and *T. cruzi*. (A) *T. cruzi* was partially tightly recovered by the macrophage plasma membrane (PM) in a process described as phagocytosis. (B) *T. cruzi* flagella recovered by host cell PM in a process described as coiled phagocytosis**. Bars = 1 μm [After Ref. (47)].

The process known as phagocytosis is a key mechanism of the innate immune response in which macrophages, dendritic cells, and other myeloid phagocytes internalize diverse microorganisms, dead or dying cells, and debris (29). Phagocytosis is an actin-dependent process that can be triggered by several types of ligands and receptors, leading to particle internalization (30). These receptors, called “pattern-recognition receptors” by Janneway (31) because of their capability to recognize pathogens, are present on the entire surface of phagocytic cells and are known as Fc receptors, complement receptors, scavenger receptors, mannose receptors, and receptors for extracellular matrix components (32). Accordingly, a classical zipper type of phagocytosis was described in addition to several unconventional phagocytic routes. In the classical zipper model, after the attachment of a pathogen to the receptor present on the host cell PM, bilateral protrusions extending from the host cell PM engulf the pathogen until a vacuole (completed sealed) is formed. Frequently, this type of phagocytosis occurs after some ligand binds to the Fc receptors or CR receptors (33). Unconventional methods of phagocytosis can be shared among three different groups according to the morphological features. The first is triggered phagocytosis, in which abundant membrane ruffles eventually enclose a spacious vacuole containing the microorganism to be ingested. This mechanism, frequently referred to as triggered macropinocytosis, is commonly driven by entero-invasive bacteria and requires a secretion of a type 3 bacteria protein complex that is responsible for translocating bacterial proteins into the host cells (34). Another unconventional mechanism is overlapping phagocytosis, which is morphologically described as forming pseudopods that do not fuse but slide past each other, resulting in pseudopod stacks to which lateral pseudopods are added. Coiling phagocytosis is characterized by the extension of unilateral pseudopods that rotate around the pathogens. Both overlapping and coiling phagocytosis are predominantly observed in professional phagocytic cells, indicating that this process is driven by the host cell (32). The signaling triggered by the pathogen varies depending on the nature of the receptors used. Basically, exposure to multivalent ligands induces clustering of these receptors in the plane of the membrane, initiating the phosphorylation of some tyrosine kinases. The remodeling of actin is unambiguously required for pseudopod extension, and in the case of FcγR, polymerization is driven by Rac1 and/or Rac2 and Cdc 42. Additionally, phosphoinositides provide an important contribution to actin remodeling during phagocytosis. Phosphatidylinositol-4,5-bisphosphate and phosphatidylinositol-3,4,5-participate in actin assembly, driving pseudopod formation. The conversion to phosphatidylinositol-3,4,5-trisphosphate is required for pseudopod extension and phagosomal closure. Phospholipases A and D have been considered essential to phagosome formation (35). With respect to the *T. cruzi* entry process, Nogueira and Cohn (36) were the first to propose that trypomastigotes enter peritoneal macrophages, L929, HeLa cell line and calf embryo fibroblasts by a phagocytic process because the treatment of these host cells with cytochalasin B (a drug that blocks the extension of actin filaments) inhibited the parasite internalization. Using cardiac muscle cells, Barbosa and Meirelles (37) demonstrated by transmission electron microscopy that trypomastigotes bind and induce a typical phagocytic process with host cell pseudopod extensions. These studies suggested the participation of endocytic mechanisms in both professional and non-professional phagocytes. In 1991, Hall et al. (38), using a macrophage cell line, described that the PV containing trypomastigotes presents CR3 receptors, β1 integrin, lysosomal membrane glycoproteins (lgp), and Fc receptors (the last only appears if trypomastigotes were previously opsonized). These results supported the hypothesis that *T. cruzi* can enter the host cell, mainly in macrophages, by phagocytosis. The recognition of Toll-like receptors 2 by trypomastigotes is also capable of inducing a phagocytic process (39) and initiating an inflammatory pathway. Additionally, several groups demonstrated the presence of PM components at the PV membrane, such as galactosyl and glycoconjugate residues (40) and sialoconjugates (41). Several signaling pathways are triggered by phagosome formation and are not different from those involved in the formation of the PV. In professional phagocytes, the activation of tyrosine kinase proteins during the initial contact with trypomastigotes was observed, followed by the recruitment of PI 3-kinase, which culminates in the polymerization of actin microfilaments and pseudopod extension. The participation of tyrosine kinases was demonstrated by Vieira et al. (42) using peritoneal macrophages treated with kinase inhibitors, such as genistein and staurosporine and this group suggested that the main process of trypomastigote entry was by phagocytosis. The participation of Rac1, Rho, and Cdc42 was also observed and will be discussed later. Currently, with new tools to study the endocytic types, the signaling pathways, and cellular components that are involved in different phagocytic mechanisms are being elucidated (macropinocytosis, CME, and participation of membrane microdomains) (5). In relation to amastigote the infection of mammalian cells seems be different when using and comparing different strains. While amastigotes from the *T. cruzi* I lineage (G strain) appears to induce phagocytosis by non-phagocytic cells (43, 44), amastigote from *T. cruzi* II as Y strain is largely phagocytized by macrophages, and occasionally by other cell types (43, 45). The amastigotes’ ability to induce phagocytosis was first demonstrated through cytochalasin D host cell’s treatment, where Procópio and colleagues (46) observed a drastic reduction of amastigotes penetration after actin polymerization inhibition. The analysis of the interaction type using these new approaches indicates that events initially described as phagocytosis may correspond to other endocytic pathways. The morphological analysis of the initial steps of *T. cruzi* invasion (trypomastigotes or amastigotes) using transmission and scanning electron microscopies revealed that this protozoan uses different mechanisms to invade host cells given that a wide type of morphological events can be observed when they are allowed to interact with the host cells. Using field emission scanning electron microscopy, we showed that even after a short interaction time, trypomastigotes, and amastigotes are ingested by peritoneal macrophages and by non-professional phagocytic cells (LLC-MK_2_). The macrophage PM can tightly recover *T. cruzi*, forming a funnel-like structure with bilateral projections of the host cell PM to internalize the parasites in a process described as a classical phagocytosis pathway, forming a long, large protrusion that recovers the parasite body, as characterized in the initial step of trigger phagocytosis (or macropinocytosis), or even forming a structure described as a coiled-coil phagosome in which the host cell PM forms coiled-coil projections (Figure 3) (47).

## Autophagy as an Inductor of Autophagosome Formation

Phagosomes can also form inside cells in a process described as autophagy. Autophagy is a self-degradative process involved in developmental regulation, the response to nutrient stress, and the clearance of damaged proteins and organelles and plays an important role in balancing sources of energy at critical times in development and in response to nutrient stress (48). Autophagy also plays a housekeeping role in removing misfolded or aggregated proteins, clearing damaged organelles, and eliminating intracellular pathogens (48). Indeed, during autophagy, intracellular membranes engulf organelles and cytoplasmic debris, and this process can be used to engulf intracellular microorganisms into a phagosome (called an autophagosome in the case of autophagy). The intracellular machinery involved in this process is complex, involving several classes of proteins, including Atg proteins (proteins related to autophagy) (49). Currently, more than 32 genes for Atg proteins have been described in mammals (49). The formation of double membrane autophagosomes also requires the activation of the mTOR protein (mammalian target of rapamycin protein) and recruitment of microtubule-associated protein light chain 3 (LC3B) and lysosome (49). This mechanism can be induced by starvation or by the use of rapamycin (which activates the mTOR pathway). Romano and colleagues (50) demonstrated that both treatments are capable of reducing the internalization of *T. cruzi* into host cells and that the PV is labeled with LC3B, a molecular marker of the autophagy pathway. Martins et al. (51) showed that treating host cells with rapamycin impairs the binding of *T. cruzi* gp82 to the host cell. This surface molecule is required for adhesion and is one molecule described to be responsible for the exocytosis of lysosomes that can lead to trypomastigote internalization (51).

## Membrane Rafts: Endocytosis Dependent on Caveolin or Flotillin

Due to their characteristic shape, caveolae have long been thought to be dynamic endocytic structures (52). In the case of mammalian cells, basically three different types of caveolin proteins are present: caveolin 1, caveolin 2, and caveolin 3 (52). Caveolin 1 and caveolin 2 are found in almost all cell types (excluding neurons and leukocytes, which do not present caveoles), and caveolin 3 mainly found in muscle cells (52). Each caveolae presents approximately 200 caveolin 1 molecules, and caveolae biogenesis is completely dependent on this protein (52). Based on this information, caveolin 1 is known to be the main caveolae marker. Caveolin 1 is also capable of binding to the GM1 ganglioside and to some GPI-anchored proteins. Cholesterol is another component of caveolae, and its depletion has been shown to promote the disorganization of the caveolar structure (53). The raft-associated proteins, flotillin 1 and flotillin 2, are also reported to play a role in endocytosis. Flotillin proteins show homology with caveolin 1, thus suggesting participation in lipid ordering (5, 54, 55). The domains that contain flotillins are morphologically distinct from caveolae because they display a flattened shape, whereas caveolae are spherical. However, both are enriched in cholesterol, GM1 and GPI-anchored proteins (55).

**Figure 4:**
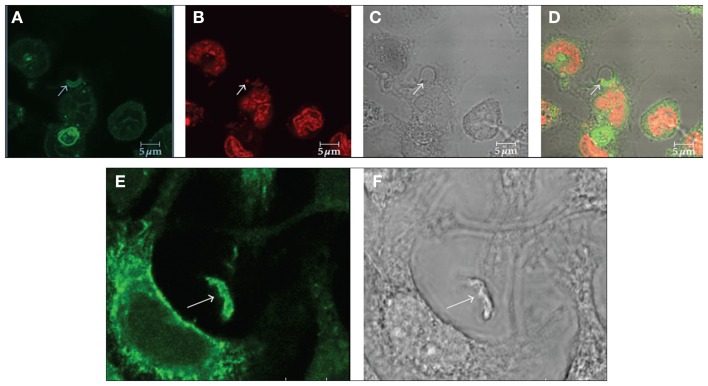
**Immunofluorescence microscopy localization of GM1 (A–D) and flotillin 1 (E,F) during internalization of *T. cruzi* by macrophages suggests the participation of membrane microdomains in this process**. **(A–D)** Co-localization of GM1, using cholera toxin subunit B **(A)** and an intracellular parasite **(C**: arrow). **(B)** Shows labeling of the nucleus and kinetoplast with propidium iodide. Corresponds to a DIC image; **(D)** is a merge image. **(E,F)** Co-localization of flotillin 1 **(A)**, detected using a specific antibody, and trypomastigotes **(B**: arrows). Bars – 5 μm. After Ref. (57).

The host cell PM microdomains have been shown to be involved in *T. cruzi* entry in both non-phagocytic and phagocytic cells (56–58). Fernandes et al. (56) and Barrias et al. (57) showed that cholesterol, the major component of membrane rafts, is involved in the *T. cruzi* entry process because the treatment of the host cells with drugs that remove or immobilize this component, such as beta cyclodextrin and filipin, impairs parasite internalization. We do not know yet if cholesterol is a direct participant in this recognition process or if the alterations caused by its removal or immobilization lead to membrane composition alterations that hide or remove receptors involved in this important process. Previously, Hissa et al. (58) showed that cholesterol depletion reduces *T. cruzi* penetration because lysosome exocytosis became unregulated after this treatment, impairing the release of acid sphingomyelinase from the lysosome, which induces endocytosis. During parasite internalization by the host cell, molecular markers of both types of membrane rafts, such as flotillin 1, caveolin 1, and GM1, were observed at the parasite-host cell PM interface (Figure 4) (57). These suggest the participation of microdomains in *T. cruzi* internalization by the host cells.

## Macropinocytosis as Another Route to *T. cruzi* Penetration

**Figure 5:**
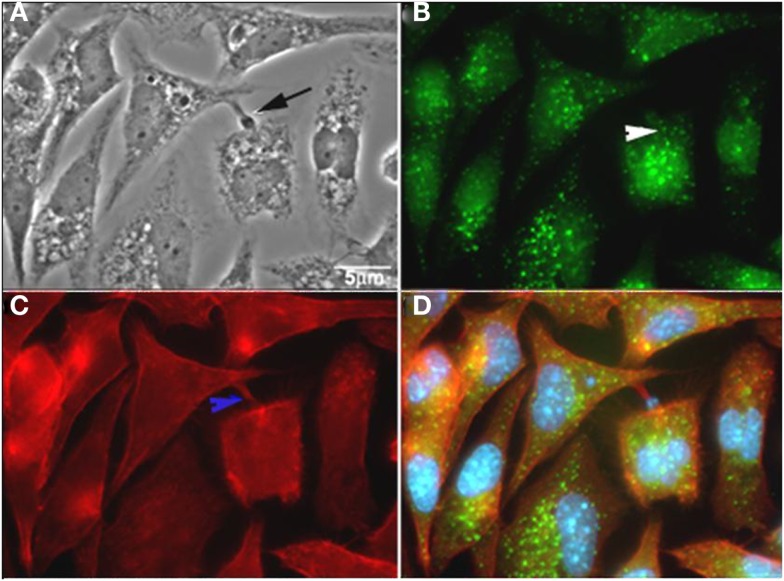
***T. cruzi* co-localizes with rabankyrin 5 and actin**. **(A)** Phase contrast; **(B)** rabankyrin 5-Alexa 488; **(C)** phalloidin-Alexa 546; **(D)** merge (rabankyrin 5, phalloidin, and DAPI). Arrow indicate trypomastigotes, white arrowhead indicates rabankirin labeling around parasites and blue arrowhead indicates host cell actin around parasites. After Ref. (62).

Macropinocytosis represents a regulated form of endocytosis that mediates the non-selective uptake of solute molecules, nutrients, antigens, and some pathogens, such as viruses. This process of endocytosis was originally described as involving the assembly of large extensions of the PM (59). The molecular basis for the formation and maturation of macropinosomes has only recently begun to be defined. Macropinocytic events may begin with external stimuli that trigger the activation of tyrosine kinase receptors, inducing changes in the dynamics of actin filaments, which then leads to PM ruffling. The Ras GTPase superfamily plays an important function in the activation process (60). After activation of the tyrosine kinase receptor, three different signaling pathways are triggered, involving the proteins Rac1, Rabankyrin 5 (an effector of Rab5 protein), Arf6, PI3K, and p21-activated kinase Pak1 (activates Rac1) (61). Rabankyrin 5 has been used as a molecular marker to distinguish macropinosomes from other endocytic compartments (61). In addition, this mechanism is also characterized by the actin-dependent reorganization of the PM to form macropinosomes, which are morphologically heterogenic vesicles that lack coat structures. Na^+^/H^+^ exchangers have also been described to play an important role in the maintenance of a macropinocytic event. Indeed, drugs that inhibit these exchangers, such as amiloride and EIPA, are widely used to characterize macropinocytosis (60). Although PI3K, Rac, and Cdc 42 have already been described as proteins involved in *T. cruzi* entry into different cell types, Barrias and colleagues (62) recently showed, for the first time, the participation of this pathway in the internalization of trypomastigotes and amastigotes of *T*. *cruzi* into phagocytic and non-phagocytic cell types. The intense inhibition of the parasite internalization process occurred when the host cells were pre-treated with amiloride (an inhibitor of Na^+^/H^+^ exchangers) or with rottlerin (an inhibitor of PKC). Host cell treatment with PMA, a stimulator of macropinocytosis caused by PKC stimulation, promotes an increase in parasite internalization. The recruitment of phosphorylated proteins, actin, and Rabankyrin 5 to the site of parasite entry and the characteristic morphology of this process, as shown by fluorescence microscopy, support the view that macropinocytosis is another process used by *T. cruzi* to penetrate host cells (Figure 5) (62). Morphologically, the entry of trypomastigotes and amastigotes in peritoneal macrophages closely resembles the process described for macropinocytosis, where there are extensive unilateral extensions of the PM that result in a loose vacuole around the parasite (62).

## Clathrin-Mediated Endocytosis

Clathrin-coated vesicles are formed during receptor-mediated endocytosis and organelle biogenesis at the trans-Golgi network (5). The clathrin coat itself is formed by the self-assembly of triskelion-shaped molecules composed of three clathrin heavy chains and associated clathrin light chain subunits (63). The diversity of the cargo and diversity of the adaptor and accessory proteins used to implement vesicle formation reflect the pathways’ adaptations to tools suited to the materials being packaged. Some well-known cargoes that use CME are tyrosine receptor kinase, GPCRs, transferrin receptor, LDL receptors, and anthrax toxin (64). Clathrin is also required for the internalization of large structures, such as bacteria (65), fungi hyphae (66), and large viruses (67), in a process that involves cooperation with actin. Recently, Nagajyotic and colleagues (68) demonstrated that the low-density lipoprotein receptor (LDLr) is important in the invasion and subsequent fusion of the PV containing *T*. cruzi with host cell lysosomes, thus suggesting the participation of clathrin-coated pits in parasite internalization because LDL receptors are concentrated in this vesicle. This demonstration was performed using an antibody against the clathrin light chain by immunofluorescence. Although this labeling was clearly observed around the vacuole, further studies should be conducted to demonstrate that the labeling is actually clathrin from the endocytic-coated vesicles and not from another cell site.

## Endo-Lysosome Participation in *T. cruzi* Invasion

After the cargo binds to mammalian cell receptors and its internalization by different endocytic pathways culminating in the activation of many signaling events, the cargo is delivered to heterogeneous organelles known as early endosomes. These organelles are usually complex presenting long thin tubules connected to bulbous or vacuolar elements and pH 6.5–6.0. Early endosomes contain molecular markers, such as the Rab5 and EEA1 proteins (“early endosome antigen”), in their membranes. The tubules are responsible for molecular sorting and vesicle transport to the endoplasmic reticulum, PM, trans-Golgi network, and other destinations (69). This organelle loss tubular elements and matures, transforming in a late endosome. The maturation is marked by the switch of molecular marker Rab5 to Rab7 (70). The late endosome displays a vesicular appearance and moves through cellular microtubules in the minus direction, allowing it to occupy a perinuclear position. In addition to Rab7, late endosomes present Rab9, Cd63, and the mannose-6 phosphate receptor (70). The Lamp1 and Lamp2 proteins, which protect the organelle from acid hydrolases, are acquired during this maturation process through fusion with the lysosome in a coordinating system that culminates in an organelle containing many vesicles inside (multivesicular bodies) and with a low pH range (4.5–5.0). The participation of early and late endosomes in the *T*. *cruzi*-host cell interaction was first characterized by Wilkowsky and colleagues (71) when they demonstrated the recruitment of Rab5 and Rab7 to the PV containing the protozoan. Woolsey et al. (72), using a short interaction time between *T. cruzi* and non-professional phagocytic cells, showed that 50% or more of the invading *T. cruzi* trypomastigotes use the host cell PM during the PV formation. They suggested that this process was facilitated by host cell actin depolymerization and showed that this vacuole is enriched in products from PI 3-kinase and that it is negative for lysosomal markers. Approximately 20% of *T. cruzi-*containing vacuoles were positive for EEA1 and Rab 5, and approximately 20% were positive for Lamp1, a lysosome marker. Since 1994 (73), the exocytosis of lysosomes to the parasite site of entry has been described as playing an important role in parasite entry. Lysosomes are placed in the path of the host cell PM along microtubules in a kinesin-dependent method (74). They fuse with the PM in a Ca^2+^-dependent process (75). This process was described as the unique way the parasite used to enter and be kept inside the host cell. However, this process was subsequently shown to represent only approximately 20% of the parasite entrance, and a lysosome-independent process was described to account for approximately 50% of the parasite internalization process. In addition, in approximately 20% of the internalized parasite, there was participation of the early endosomes, as recognized by EEA1 labeling 10 min post-infection (72). They also described that the PV vacuole containing *T. cruzi* was observed labeled with the lysosome associated protein 1 (LAMP1) as well as with endocytic tracer from pre-labeled lysosomes. These results showed that the lysosome pathway was not the only one that presents fusion with lysosomes. Barr et al. (76) showed that an unusual 120-kDa alkaline peptidase (TSF) from a soluble fraction of *T. cruzi* induces repetitive calcium transients in primary isolated cardiac myocytes from dogs. Using thapsigargin, they also showed that Ca^2+^ depletion from intracellular stores, such as the sarcoplasmic reticulum, is able to inhibit Ca^2+^ transients and trypomastigote invasion. The authors also described that “the Ca^2+^ transients are dependent on release of Ca^2+^ from sarcoplasmic reticulum Ca^2+^ stores, but this release in not dependent on extracellular Ca^2+^ or on the classic model of Ca^2+^-induced Ca^2+^ release in cardiac myocytes.” In 1999, Meirelles et al. (77) also described that the sarcoplasmic reticulum Ca^2+^ ATPase (SERCA) participates in trypomastigote invasion into cardiomyocytes because thapsigargin inhibits 75% of this process. Recently, Fernandes et al. (78) showed that the entry of *T. cruzi* trypomastigotes into the host cell wounds the host cell PM by inducing a process of wound repair using Ca^2+^-dependent exocytosis of lysosomes. The lysosome exocytosis was triggered by an increase in calcium influx, derived from the extracellular space, which enters the host cell as soon as the PM is wounded. The wound repair of the host cell PM was performed with the lysosomal delivery of acid sphingomyelinase to the host PM and formation of endosomes enriched in ceramide, processes that facilitate parasite entry into the host cell (78). Besides, this mechanism may be involved with the tropism of *T. cruzi* for cardic cells since membrane repair is common in muscle cells, explaining part of the Chagas’ disease pathology (78).

## Actin Cytoskeleton

The participation of the actin cytoskeleton during the initial step of the invasion has, until now, been under debate. The participation of the actin cytoskeleton in *T*. *cruzi* entry has been suggested since 1976 when Dvorak and colleagues (79) treated different host cells with cytochalasin B and demonstrated unequivocally that the internalization of trypomastigotes was impaired. Rosestolato et al. (80), using different host cells (professional and non-professional phagocytic cells) previously treated with cytochalasin D (CD) and then allowed to interact with the cell culture trypomastigote forms, also showed a drastic reduction of the parasites inside the host cells (81). Additionally, Barbosa and Meirelles (37), using heart muscle cells, clearly showed the evident participation of the actin cytoskeleton during *T. cruzi* invasion. In 2004, Woolsey and Burleigh (72) showed that actin depolymerization by cytochalasin D enhances parasite entry into the host cell at an early step and also blocks lysosome or early endosome fusion at the site of parasite entry. They also described, using NIH-3T3 fibroblasts expressing dominant-negative Rho, that after 15 min of infection, that there were three times more parasites inside than in the control cells but that the number of intracellular parasites drastically decreased until 1 h. They suggested that a cell with continuous actin cytoskeleton alterations was not able to retain the parasites inside the cell, showing the importance of actin polymerization and depolymerization on the interaction process. Our group showed (82) that cells overexpressing Rac 1 exhibited a higher internalization index for *T*. *cruzi* compared with normal cells. However, after 48 h, a reduced number of parasites were observed. Notably, these different results can be explained by different host cell treatments, whether the cells were washed after the incubation with cytochalasin, the interaction time after the drug treatment, the nature of the parasite strain, and other considerations. We also believe that despite the contradictory results, all these papers contribute to a better understanding of the complex process of the *T. cruzi*-host cell interaction and that it is not good scientific practice to neglect a thorough discussion of all published results, as frequently happens.

During the initial moments of the interaction process with *T. cruzi* trypomastigotes, the host cell transient calcium increase has been reported (73–76). Host cells treated with thapsigargin, an inhibitor of endoplasmic reticulum Ca^2+^-ATPase (83) that reduces parasite entry into the host cell (84), showed the participation of the intracellular calcium store in this process and its involvement in lysosome exocytosis.

## Parasitophorous Vacuole’ Closure

In mammalian cells, several molecules that selectively regulate the assembly of an endocytic vacuole have been identified. Among them, dynamin has been shown to play a major role in processes such as CME, synaptic vesicle recycling, phagocytosis, transport from the trans-Golgi network, and ligand uptake through caveolae (85). Dynamin is a GTPase family comprising three isoforms: dynamins 1, 2, and 3 (86). One protein class that interacts with dynamin is phosphatidylinositol-3-kinase (PI3K) (87). Dynamin interacts with the p85 regulatory subunit of PI3K, and this interaction stimulates the GTPase activity of dynamin. Gold and colleagues (88) reported that the inhibition of PI3K prevents the recruitment of dynamin 2 to the site of particle binding, suggesting that in phagocytosis, the activation of PI3K is upstream of dynamin. According to some models, dynamin is a mechanochemical enzyme that is directly responsible for pinching off the vesicle (86). Other authors consider that dynamin is a regulatory protein that recruits the downstream partner, which, in turn, drives the fission step (87, 89). Using dominant-negative dynamin (K44A) HeLa cells, Wilkowsky and colleagues (71) showed that dynamin is involved in the invasion of *T. cruzi* in non-phagocytic host cells. Subsequently, Barrias et al. (62) showed that the GTPase activity of this protein is important for the fission of PVs in both phagocytic and non-phagocytic cell lines through the use of dynasore, which has the ability to block the GTPase activity of dynamin, acting as a potent inhibitor of endocytic pathways by blocking coated vesicle formation within seconds of its addition.

## Concluding Remarks

More than 100 years after Carlos Chagas’ discovery about *T. cruzi* and Chagas’ disease, we still have many important gaps in the knowledge of the basic aspects of the protozoan biology and its interaction with host cells. It is now clear that the parasite uses several surface-associated molecules to interact with a not yet completely defined set of macromolecules exposed on the host cell surface. We also now know that several internalization processes are triggered following the parasite ligand-host cell receptor interactions. However, we still do not know which ligand-receptor complex triggers each of the types of internalization. New methods and approaches are necessary to better understand the parasite-host cell interaction process. The use of parasite molecules to recover latex beads and their use to interact with host cells may provide new information as to how different parasite molecules could act in the parasite-host cell junction. Unfortunately, neither gene knock-out nor gene silencing is effective with *T*. *cruzi*. The use of other methodologies, such as high-throughput technology, gene knock-out of host cell molecules by RNAi and microarray platforms, can provide new insights into this fascinating field of research. Altogether, it is clear that now we have much more information on the process of interaction of *T. cruzi* with host cells, especially the various mechanisms the parasite uses to penetrate into host cells. It is now important to identify the key molecules involved on each process and develop drugs able to inhibit the infection of the cells by the parasite, opening a new approach to the treatment of the acute phase of Chagas disease, where amplification of the infection through successive invasion of the cells plays a fundamental role.

## Conflict of Interest Statement

The authors declare that the research was conducted in the absence of any commercial or financial relationships that could be construed as a potential conflict of interest.
